# Threatened abortion and its consequences on maternal and fetal health

**DOI:** 10.6026/973206300214499

**Published:** 2025-12-15

**Authors:** Salma Akter Munmun, Rowson Ara, Sabiha Islam, Farah Noor, Reefaat Rahman, Sayada Fatema Khatun, Shahinoor A.M

**Affiliations:** 1 Department of Obstetrics and Gynecology, Bangladesh Medical University (BMU), Dhaka, Bangladesh, South Asia; 2 Department of Gynecological Oncology, Bangladesh Medical University (BMU), Dhaka, Bangladesh, South Asia; 3 Department of Pediatric Surgery, Bangladesh Medical University (BMU), Dhaka, Bangladesh, South Asia

**Keywords:** Threatened abortion, maternal complications, fetal outcomes, preterm delivery, low birth weight, stillbirth and cohort study

## Abstract

Threatened abortion, defined as vaginal bleeding in the first trimester of pregnancy with a viable fetus, is a common obstetric complication. Therefore, it is of interest to investigate the maternal and fetal health outcomes associated with threatened abortion and to compare these outcomes with a control group of healthy pregnancies. Hence, a total of 240 pregnant women were included in this study, with 120 women diagnosed with threatened abortion and 120 healthy controls. Women with threatened abortion showed significantly higher rates of preterm delivery (25% vs. 12.5%, p<0.001), low birth weight (23.33% vs. 10%, p<0.001) and intrauterine growth restriction (12.5% vs. 6.67%) compared to the control group. Additionally, the incidence of antepartum hemorrhage (9.17% vs. 3.33%, p=0.001) and postpartum hemorrhage (10% vs. 4.17%) were significantly higher in the threatened abortion group. Neonatal outcomes also showed higher rates of low Apgar scores (10% vs. 3.33%, p<0.001) and NICU admissions (28.33% vs. 6.67%). Our study highlights the significant risks associated with threatened abortion, including increased rates of preterm birth, low birth weight, fetal growth restriction and maternal complications. These findings emphasize the need for early identification and careful management of pregnancies complicated by threatened abortion.

## Background:

Threatened abortion, commonly known as threatened miscarriage, is defined as the occurrence of vaginal bleeding during the first 20 weeks of gestation without any accompanying cervical dilation or, alternatively, as cervical dilation in the absence of vaginal bleeding during early pregnancy [1, 2]. The diagnosis is confirmed through a combination of clinical evaluation, which identifies vaginal bleeding with a closed cervical OS and ultrasonographic evidence of fetal cardiac activity [3]. Vaginal bleeding in the first trimester is a common complication, affecting 16% to 25% of all pregnancies [4]. Approximately 10-20% of all pregnancies are at risk of miscarriage and about one-quarter of all pregnancies experience hemorrhage before 20 weeks of gestation [4, 5]. On a global scale, first-trimester vaginal bleeding is a frequent occurrence, with reports indicating its presence in one-fifth of all pregnancies [6]. Threatened abortion represents a substantial percentage of hospital admissions during the early stages of pregnancy [7]. Despite its prevalence, about 85-95% of pregnancies with confirmed fetal cardiac activity on ultrasound continue beyond the 24th week of gestation [2]. However, in the absence of fetal viability, the risk of spontaneous abortion increases significantly, reaching up to 50% [8]. Endocrine and inflammatory mechanisms within the endometrium are essential for the successful progression of pregnancy, supporting processes from implantation through to delivery [9]. Any disruption in these mechanisms can result in complications with placental invasion, leading to first-trimester hemorrhage and substantially elevating the risk of miscarriage or other adverse pregnancy outcomes. Pregnancies complicated by threatened abortion are associated with increased risks of adverse outcomes such as preeclampsia, intrauterine growth restriction (IUGR), low birth weight, abruptio placentae and preterm delivery [10]. Additionally, vaginal bleeding during early pregnancy is believed to indicate underlying placental dysfunction, which may play a role in the development of these complications [11]. Meta-analyses suggest that bleeding in the first trimester doubles the risk of various complications, such as gestational hypertension, preterm rupture of membranes, placental abruption and postpartum hemorrhage. Despite these findings, approximately half of the bleeding episodes have no identifiable cause, making it challenging to predict which pregnancies will develop complications [12]. Threatened miscarriage is a known cause of significant anxiety for affected women and their caregivers. This condition contributes to an increased financial burden, as many cases require specialist consultations and interventions, such as bed rest or progesterone supplementation [13]. While several studies have explored the association between first-trimester vaginal bleeding and adverse pregnancy outcomes, the data on outcomes beyond fetal viability remain limited [11]. Therefore, it is of interest to describe the differences in pregnancy outcomes between women with and without threatened abortion.

## Materials and Methods:

This was a prospective observational study conducted Department of Obstetrics and Gynecology, Bangladesh Medical University (BMU), Dhaka, Bangladesh from July 2023 June 2024. The study was approved by the Institutional Ethics Committee and written informed consent was obtained from all participants prior to enrollment. A total of 240 pregnant women were included in the study. Participants were divided into two groups: the case group (n=120), comprising women diagnosed with threatened abortion and the control group (n=120), consisting of pregnant women with no history of vaginal bleeding during early pregnancy.

## Inclusion criteria:

The study included pregnant females with a single intrauterine pregnancy. Gestational age was determined by the patient's reported last menstrual period (LMP) and confirmed by first-trimester ultrasound. The diagnostic criteria for threatened abortion were based on documented history of vaginal bleeding with a closed cervix before 20 weeks of gestation, along with ultrasound-confirmed fetal heart activity at the time of presentation.

## Exclusion criteria:

Participants were excluded from the study if they had chronic systemic diseases such as chronic hypertension, diabetes mellitus, or thrombophilia, as these conditions could impact pregnancy outcomes. Additionally, those with a history of trauma or surgery during the current pregnancy, smoking, multiple pregnancies, or recurrent abortion were excluded. Women with congenital uterine anomalies, large leiomyomata distorting the uterine cavity, or cervical incompetence or local cervical pathologies (e.g., cervical polyps) were also not included, as these conditions could influence the pregnancy and its outcomes.

## Data collection and variables:

Demographic data, obstetric history and clinical parameters were recorded at the time of diagnosis, including maternal age, parity and gestational age at presentation. Women in the threatened abortion group during the first trimester received 200 mg of progesterone supplementation twice daily in the form of rectal suppositories until one week after the cessation of bleeding. Both the threatened abortion and control groups were scheduled for examination every two weeks to assess any adverse pregnancy outcomes. Laboratory investigations, including serum progesterone levels, complete blood count (CBC) and inflammatory markers, were measured at baseline and follow-up visits. Participants were followed until delivery and various maternal and fetal outcomes were assessed. Maternal complications, including preterm labor, placental abruption, hypertensive disorders and the need for hospitalization, were documented. Additionally, the study recorded the occurrence of preterm delivery (<37 weeks), cesarean delivery, antepartum hemorrhage, gestational diabetes mellitus and postpartum hemorrhage. Fetal outcomes, such as intrauterine growth restriction (IUGR), small for gestational age (SGA), low birth weight (<2500 g), stillbirth and neonatal intensive care unit (NICU) admission, were also documented. Neonatal outcomes were further assessed by measuring the Apgar score at 5 minutes (with a score <7 indicating possible neonatal distress) and neonatal mortality. These parameters were evaluated to determine the impact of threatened abortion on both maternal and fetal health throughout the pregnancy.

## Statistical analysis:

Data were analyzed using SPSS version 26.0. Descriptive data were presented as frequencies and proportions. Quantitative data were presented as means ± standard deviation (SD). The independent-samples Student's t-test was used for normally distributed variables, while the Mann-Whitney U test was applied for non-normally distributed variables. Pearson chi-square tests were used to assess differences in categorical variables. Logistic regression was computed to assess the relationship between dependent variables (pregnancy complications) and independent variables (sub-groups). A p-value of <0.05 was considered statistically significant.

## Results:

A total of 240 participants were included, with 120 in the threatened abortion group and 120 in the control group. The mean maternal age was slightly lower in the threatened abortion group (26.05±5.90 years) compared to the control group (26.9±5.5 years), but the difference was not significant. Most participants were aged 30-39 years (52.5% in the threatened abortion group, 57.5% in the control group). Maternal BMI was similar between groups (23.12±3.5 kg/m^2^ in the threatened abortion group vs. 23.5±2.9 kg/m^2^ in the control group, p=0.51). Nulliparity was more frequent in the control group (35.83%) than in the threatened abortion group (29.17%), while multiparity was higher in the threatened abortion group (36.67%) than in the control group (25.83%) (Table 1 - see PDF). Table 2 (see PDF) showed that pregnancy outcomes differed significantly between the groups. Preterm delivery (<37 weeks) was observed in 25.00% of the threatened abortion group and 12.50% of the control group, with an adjusted odds ratio (AOR) of 2.31 (95% CI: 1.12-4.78, p<0.001). Low birth weight (<2500 g) was recorded in 23.33% of the threatened abortion group and 10.00% of the control group (AOR: 2.69, 95% CI: 1.24-5.82, p<0.001). Small-for-gestational-age neonates were noted in 16.67% of the threatened abortion group and 8.33% of the control group, though this difference was not statistically significant (AOR: 2.19, 95% CI: 0.95-5.03, p=0.065). Maternal complications were more common in the threatened abortion group.

Antepartum hemorrhage occurred in 9.17% of the threatened abortion group and 3.33% of the control group (p=0.001). Postpartum hemorrhage was observed in 10.00% of the threatened abortion group and 4.17% of the control group (p=0.001). Hypertensive disorders of pregnancy were found in 19.17% of the threatened abortion group and 10.00% of the control group (p=0.061). Gestational diabetes mellitus was present in 15.00% of the threatened abortion group and 8.33% of the control group (p=0.186) (Table 3 - see PDF). A low Apgar score (<7) at 5 minutes was recorded in 10.00% of neonates in the threatened abortion group and 3.33% in the control group (p<0.001). NICU admission was required in 28.33% of neonates from the threatened abortion group and 6.67% from the control group. Neonatal mortality was reported in 5.00% of the threatened abortion group and 1.67% of the control group (Table 4 - see PDF).

## Discussion:

Pregnancy-related bleeding presents a significant clinical challenge and is closely associated with substantial maternal and fetal morbidity [14]. Threatened abortion, defined by vaginal bleeding within the first 20 weeks of pregnancy with a closed cervical OS, affects approximately 15-20% of all pregnancies [15]. The likelihood of adverse pregnancy outcomes, including abortion, increases with both the volume and duration of bleeding, especially when accompanied by lower abdominal pain [14]. These outcomes contribute to the elevated morbidity observed in both mothers and fetuses [16]. In our study, we evaluated the impact of threatened abortion on maternal and fetal outcomes and observed a significant association with adverse pregnancy complications. The mean maternal age was 26.05 years in the threatened abortion group and 26.9 years in the control group, with no statistically significant difference observed (p = 0.068). Similarly, a study by Amirkhani *et al.* reported that 53% of participants were aged between 25 and 34 years, with no significant difference in age distribution between the groups (p = 0.34) [17]. The BMI of the participants in both groups was also similar, with mean values of 23.12 and 23.5 kg/m^2^, respectively and no significant difference (p = 0.51). Regarding parity, there was no significant difference between the groups in terms of nulliparity, primiparity and multiparity (p = 0.139). These results align with the findings of Sammut *et al.* who also reported no association between maternal age or BMI and the risk of threatened abortion [18]. Consistent with our study, Agarwal *et al.* documented similar mean ages of 23.85±3.48 years in the study group and 23.78±3.07 years in the control group, with corresponding mean BMIs of 20.67±1.26 kg/m^2^ and 20.53±1.43 kg/m^2^ [16, 19]. A significant finding of our study was the higher incidence of preterm delivery among women with threatened abortion, observed in 25.00% of cases compared to 12.50% in the control group (p < 0.001). This finding aligns with previous research, including a cohort study by Patel *et al.* which also reported a strong association between first-trimester bleeding and an increased risk of preterm birth [14]. However, in contrast, a study by Strobino *et al.* did not establish a significant link between threatened abortion and preterm delivery [20]. The underlying pathophysiology of this association may involve placental dysfunction and inflammatory processes, both of which have been implicated in the mechanisms leading to premature labor [16]. Our study revealed a significant association between threatened abortion and low birth weight, with the threatened abortion group showing a higher incidence (23.33%) compared to the control group (10.00%) (p < 0.001).

A similar finding was reported by Ghosh *et al.* in their study [21]. Previous research has suggested that first-trimester bleeding, often associated with placental disorders, is linked to a shorter gestational period and an increased risk of premature delivery. These factors contribute to intrauterine growth restriction, leading to lower birth weights in newborns due to prematurity [22]. Intrauterine growth restriction (IUGR) was observed in 12.50% of cases in the threatened abortion group, which was significantly higher than the 6.67% observed in the control group. This finding is consistent with the report by Ghosh *et al.* who documented an IUGR rate of 8.10% in women with threatened abortion [21]. The cesarean delivery rate was higher in the threatened abortion group (41.67%) compared to the control group, but this difference was not statistically significant (p = 0.121). Agarwal *et al.* reported similar findings, with no significant difference in cesarean rates between the case (32.65%) and control groups (28.85%, p = 0.84) [16]. Maternal complications were more prevalent in the threatened abortion group. Antepartum hemorrhage (APH) was observed in 9.17% of women with threatened abortion, aligning with previous research that has demonstrated an elevated risk of APH in this population [11]. Moreover, postpartum hemorrhage (PPH) was significantly higher in the threatened abortion group (10.00% vs. 4.17%, p = 0.001). Furthermore, postpartum hemorrhage (PPH) was observed more frequently in the threatened abortion group (10.00% vs. 4.17%, p = 0.001), potentially due to the increased likelihood of placental abnormalities or uterine atony associated with these pregnancies. However, no significant association was found for other maternal complications, including hypertensive disorders of pregnancy and gestational diabetes, which mirrors the results of Patel *et al.* who also reported similar complications without statistical significance. Neonatal outcomes further reflected the adverse effects associated with threatened abortion. Apgar scores below 7 at 5 minutes were more frequent in the threatened abortion group (10.00%) compared to the control group (3.33%) (p < 0.001) and NICU admissions were notably higher (28.33% vs. 6.67%; p < 0.001). These findings are in line with previous studies that have reported increased NICU admissions among infants born to mothers who experienced threatened miscarriage [14]. Limitations of this study include its retrospective design which may introduce selection bias due to potential inaccuracies or incompleteness in medical records. Additionally, the single-center setting limits the generalizability of the results to other populations. While we adjusted for factors like maternal age and BMI, other confounders, such as socioeconomic status or lifestyle, were not considered, potentially affecting outcomes. The lack of long-term follow-up is another limitation, as we only examined immediate pregnancy and neonatal outcomes. Finally, the reliance on medical records may lead to data quality issues, affecting the accuracy of reported outcomes. Future prospective, multicenter studies are needed to address these limitations. Future research should focus on multi-center studies to better understand the pathophysiology of threatened abortion and evaluate potential interventions to improve maternal and fetal outcomes.

## Conclusion:

Threatened abortion was associated with significantly increased risks of preterm delivery, low birth weight and intrauterine growth restriction in our study. Data shows the clinical relevance of early detection and close monitoring in pregnancies complicated by threatened abortion. The study also identified higher rates of maternal complications, such as antepartum and postpartum hemorrhage, emphasizing the need for vigilant care in these high-risk cases.
